# Spontaneous Reattachment of a Posteriorly Dislocated Endothelial Graft: A Case Report

**DOI:** 10.1155/2013/631702

**Published:** 2013-03-03

**Authors:** Ka Wai Kam, Alvin L. Young

**Affiliations:** Department of Ophthalmology and Visual Sciences, The Chinese University of Hong Kong, Prince of Wales Hospital, Shatin, Hong Kong

## Abstract

A thirty-year-old Chinese man with a history of severe trauma to his right eye, with secondary sectoral aniridia and multiple operations including intraocular lens insertion more than fifteen years ago, underwent an uneventful Descemet's Stripping Automated Endothelial Keratoplasty (DSAEK) for his pseudophakic bullous keratopathy in a tertiary hospital in Hong Kong. The nature of his previous operations was unknown to the surgeon at the time of transplant. On postoperative day one, the graft was not present in the anterior chamber. Fundal view was limited because of corneal oedema. B-scan ultrasonography could not detect any definite presence of a donor button in the posterior segment as gas was present in the vitreous cavity. The patient was instructed to lie prone full time, and on postoperative day three, the graft was found to be reattached to the stroma with spontaneous resolution of corneal oedema, indicating restoration of pump function of endothelium graft. This is the first case of spontaneous reattachment of a posteriorly dislocated endothelial graft without surgical intervention or abandonment of the grafted endothelial button.

## 1. Case Report

A thirty-year-old Chinese man with a history of severe trauma to his right eye more than fifteen years ago and multiple operations done was scheduled for DSAEK for his pseudophakic bullous keratopathy in June 2012. As the patient could not recall the timing and nature of the procedures he had previously undergone, his archived old medical records were also not available, and hence details of his previous operations were not known to the operating surgeon at the time of transplantation. The patient could not recall whether his intraocular lens was scleral-fixated or a normal posterior capsule intraocular lens. Preoperative visual acuity was 20/200 and examination found pseudophakic bullous keratopathy with traumatic aniridia from 9 to 6 hours. No conjunctival bleb was seen. One faint subconjunctival suture was seen at 4 o'clock with the presence of an intraocular lens.

The patient had surgery under retrobulbar anaesthesia. DSAEK button of 8 mm was prepared with a Barron Donor Cornea Punch. After temporal peritomy, a temporal scleral tunnel 6 mm was prepared. The Descemet's membrane was stripped (and edges of stroma roughened) and later was removed through the limbal tunnel in a viscoelastic filled anterior chamber (AC). The AC was then flushed thoroughly with the presence of an infusion cannula as AC maintainer. ACIOL plastic glide was inserted into AC, and the DSAEK button was pulled into the eye by Tan's forceps (Asico, Westmont, USA). Secure and good graft apposition was achieved on table by AC air fill to ~30/40 mmHg for 5 minutes. Stab fenestrations were made for interface fluid release. At the end of the operation, the posterior lenticule was well centred and supported by full chamber gas fill of the anterior chamber. The patient was instructed to lie supine full time postoperatively. 

**Figure 1:**
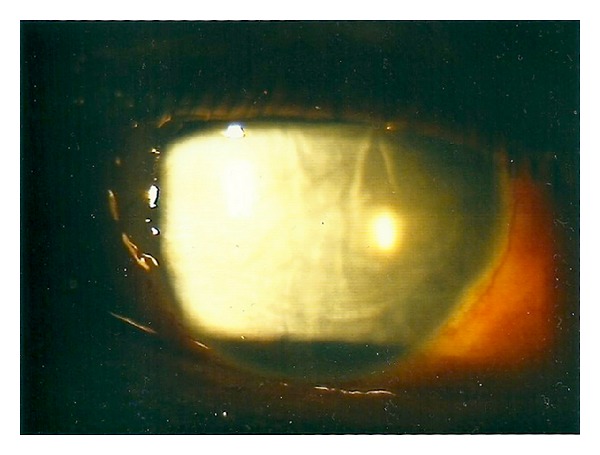
Corneal oedema without EK button.

**Figure 2:**
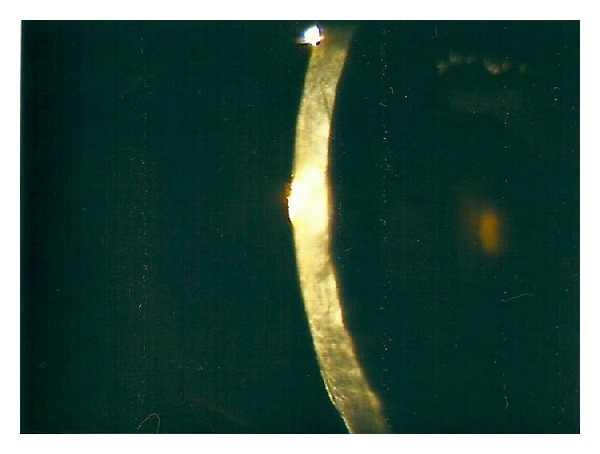
Cross-section showing the absence of EK button on post-operative day 2.

On postoperative day one, the cornea was oedematous (Figure 1), and the graft lenticule was not present in the anterior chamber (Figure 2). No gas bubble was seen in the anterior chamber. Stromal oedema limited the fundal view and a B-scan ultrasonography could not detect any definite presence of a donor button in the posterior segment as gas was present in the vitreous cavity. 

**Figure 3:**
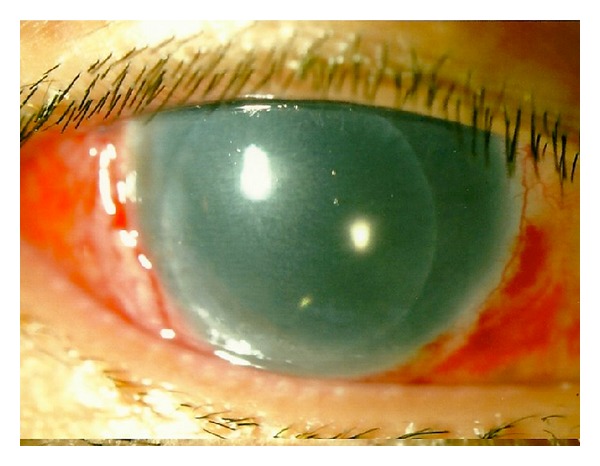
Reappearance of EK button (slightly decentered) and resolution of corneal oedema.

**Figure 4:**
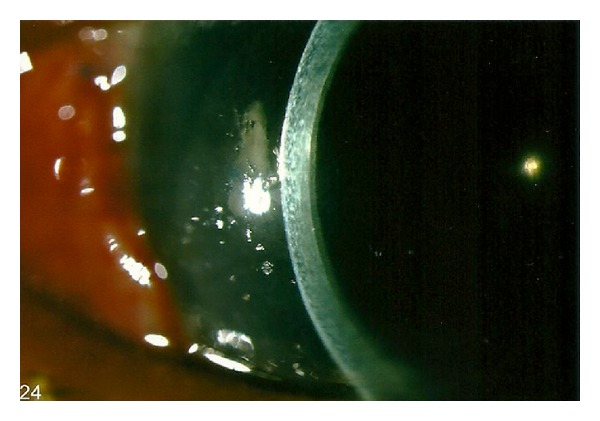
Well-opposed EK button and clear recipient cornea.

The patient was instructed to lie prone full time except for the application of topical corticosteroids and antibiotics for the following two days. On postoperative day three, the patient reported a marked improved clarity of vision since wakening. Slit-lamp examination revealed a decentered EK button over the inferonasal quadrant of the cornea (Figure 3). The best-corrected visual acuity was 20/50. Corneal oedema had largely subsided, and the graft was well opposed to the stromal bed (Figure 4). The graft-stroma interface was clear, and the inferior edge appeared secure. In view of his relative good vision, uncertain prognosis, and multiple operations (subsequently revealed a history of scleral fixation IOL) from his previous major trauma, conservative measure was adopted rather than for further graft centration adjustment and the patient was kept in prone position with his head down over the following postoperative week. 

**Figure 5:**
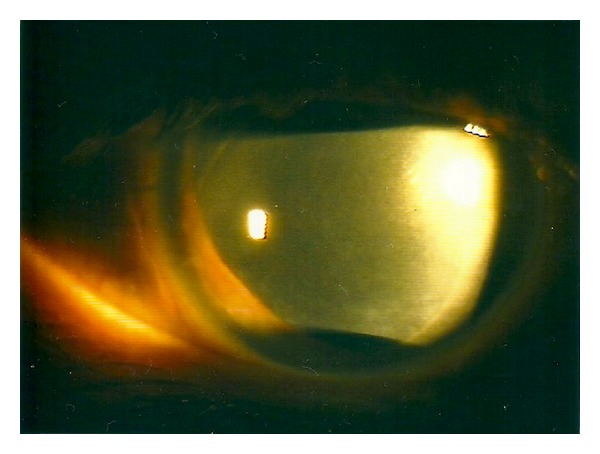
Slightly inferiorly displaced EK button and clear recipient cornea.

**Figure 6:**
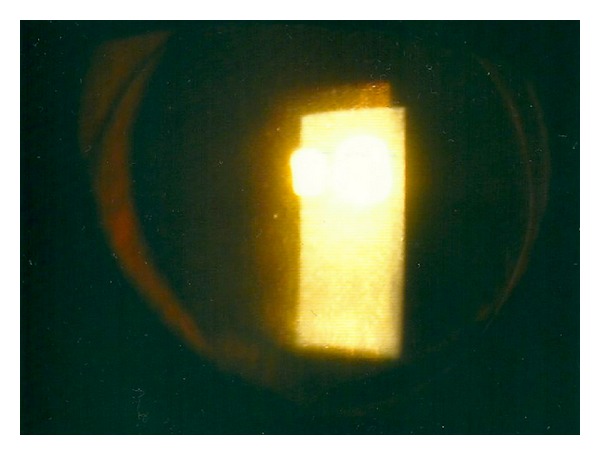
Slightly inferiorly displaced EK button and clear recipient cornea.

At his latest followup in December 2012, his visual acuity was stable at 20/30 with a healthy clear cornea and a securely attached endothelial graft (Figures 5 and 6). Specular microscopy was attempted many times but was not recordable due to the marked IOL reflection in an aniridic eye. 

## 2. Discussion

Descemet's Stripping Automated Endothelial Keratoplasty (DSAEK) is a recent technique in lamellar keratoplasty, which replaces only the posterior lamella of a diseased cornea and is considered an alternative to penetrating keratoplasty for posterior corneal diseases. The main indications include pseudophakic bullous keratopathy, Fuch's endothelial dystrophy, failed penetrating keratoplasty, or iridocorneal syndrome. And unlike penetrating keratoplasty, DSAEK offers a faster healing time and a more predictable refractive outcome as no sutures are placed on the cornea [1, 2]. Many variations in the surgical techniques were described particularly on the delivery of the posterior lenticule into the anterior chamber, and methods of securing the endothelial graft with either gas injection or anchoring suture were described to facilitate successful surgery [3, 4]. 

Graft dislocation into the posterior segment of an eye during the early postoperative period is a known but thankfully rare complication of DSAEK, occurring in only 8 out of more than 1300 DSAEK procedures [5]. The risk of dislocation into the posterior segment is higher in eyes with aniridia or eyes that had undergone vitrectomy, complicated intraocular lens implantation, or glaucoma surgery [6, 7] or had a history of trauma. All of these factors were subsequently revealed to be present in our case. 

Owing to the risks of further complications arising from the posteriorly dislocated grafts, such as retinal detachment [8], cystoid macular oedema, and epiretinal membrane formation, the dislocated grafts in all previously reported cases were retrieved either in the same operation or later by either a standard three-port vitrectomy or an anterior approach with irrigation and aspiration through the corneal wound. Histopathological studies of the retrieved grafts found significant hypocellularity, and occasionally, inflammatory cells were found to be adhered to the donor lenticule, signifying the presence of inflammation and guarded viability of the dropped graft despite successful retrieval [9]. Hence, most patients required a repeat DSAEK or penetrating keratoplasty after retrieval and abandonment of the original EK graft in order to regain visual acuity. The authors recommended retrieval of dropped graft as soon as possible, but the exact timing was not discussed at length.

To the best of our knowledge, spontaneous reattachment of posteriorly detached endothelial graft has not been reported in the literature. Although there was a recent series of spontaneous reattachment of endothelial grafts which were dislocated partially or free floating in the anterior chamber [10], no reports had been made of spontaneous reattachment of an endothelial button that was completely dislocated into the posterior segment. Our patient was the first case reported to have a full functioning reattached graft, solely by gravity and positioning, without the need for a secondary retrieval or need of abandonment of the retrieved graft. Rapid resolution of the cornea oedema indicated good endothelial function after its spontaneous reattachment. Despite its slight decentration, the graft remained stable in position, and postoperative visual acuity returned to 20/30 at the six-month followup period. Conservative prone positioning in posterior dislocated endothelial grafts may be a worthwhile measure for consideration.
